# Giant Rectus Sheath Hematoma: Pseudobladder Sign

**DOI:** 10.31662/jmaj.2023-0028

**Published:** 2023-05-22

**Authors:** Hiroshi Shiba, Tomomi Endo, Hirohisa Fujikawa, Tatsuo Tsukamoto

**Affiliations:** 1 Department of Nephrology and Dialysis, Medical Research Institute KITANO HOSPITAL, PIIF Tazuke-Kofukai, Osaka, Japan; 2 Department of Internal Medicine, Suwa Central Hospital, Nagano, Japan; 3 Department of Medical Education Studies, International Research Center for Medical Education, Graduate School of Medicine, The University of Tokyo, Tokyo, Japan

**Keywords:** rectus sheath hematoma, COVID-19, anticoagulation therapy, ultrasound

A 75-year-old woman with coronavirus disease 2019 (COVID-19) and on prophylactic heparin reported acute abdominal pain provoked by coughing on Day 6 of hospitalization. She developed peritoneal signs and hemorrhagic shock on Day 7. A bedside ultrasound revealed a hypoechoic fluid collection over a hyperechoic layer, which was initially thought to be a urinary bladder hematoma (Figure 1A). However, dynamic contrast-enhanced computed tomography revealed rectus sheath hematoma (RSH) with multiple areas of active extravasation (Figure 1B and 1C). The patient was conservatively treated and underwent elective percutaneous drainage with no recurrence. Interventional radiology embolization was avoided due to the risk of tissue necrosis.

**Figure 1.**
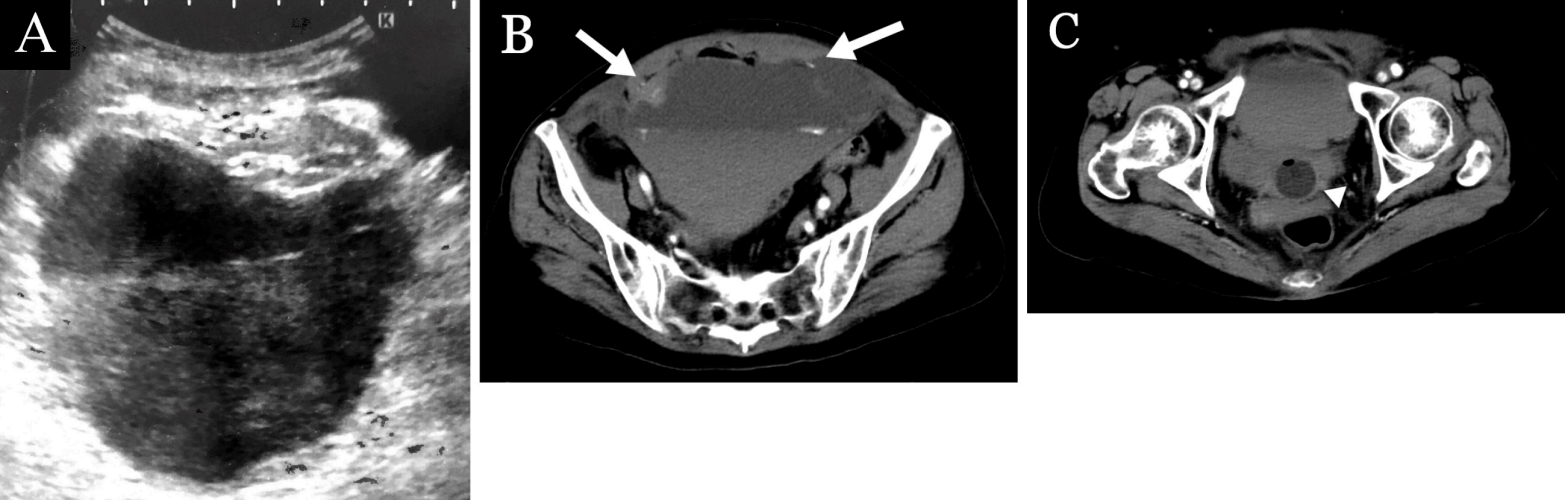
a. An ultrasound image resembling urinary bladder hematoma. b. Dynamic contrast-enhanced computed tomography (DCE-CT) showing a large hematoma in the rectus sheath, which extended below the arcuate line, with active bleeding and the hematocrit effect. There was evidence of contrast agent leakage between the rectus abdominis muscle and the hematoma. (arrows) c. A DCE-CT showing the hematoma compressing the urinary bladder, which contains a Foley catheter (arrowhead) (“pseudobladder sign”).

RSH should be suspected in COVID-19 patients with abdominal pain, as anticoagulation, cough, and COVID-19 infection itself can be risk factors for RSH ^(1), (2)^. Although ultrasound is commonly used as the first-line diagnostic modality for RSH, it may lead to misdiagnosis when it fails to detect the origin of a large hematoma ^(3)^. When the hematoma extends to the pelvic area, it may mimic bladder distension, as observed in our case. To alert clinicians of this misleading image, we propose to refer it as the “pseudobladder sign.”

## Article Information

### Conflicts of Interest

None

### Author Contributions

HS acquired data and drafted the manuscript. TE, HF, and TT reviewed and supervised the manuscript.

### Informed Consent

We have obtained informed consent for this manuscript.

### Approval by Institutional Review Board (IRB)

In this study, IRB approval was not required.
